# Individual variation in growth and physiology of symbionts in response to temperature

**DOI:** 10.1002/ece3.9000

**Published:** 2022-06-07

**Authors:** Casey P. terHorst, Mary Alice Coffroth

**Affiliations:** ^1^ Department of Biology California State University, Northridge Northridge California USA; ^2^ Department of Geology University at Buffalo Buffalo New York USA

**Keywords:** acclimation, adaptation, climate change, coral reefs, genetic variation, symbiosis

## Abstract

In many cases, understanding species’ responses to climate change requires understanding variation among individuals in response to such change. For species with strong symbiotic relationships, such as many coral reef species, genetic variation in symbiont responses to temperature may affect the response to increased ocean temperatures. To assess variation among symbiont genotypes, we examined the population dynamics and physiological responses of genotypes of *Breviolum antillogorgium* in response to increased temperature. We found broad temperature tolerance across genotypes, with all genotypes showing positive growth at 26, 30, and 32°C. Genotypes differed in the magnitude of the response of growth rate and carrying capacity to increasing temperature, suggesting that natural selection could favor different genotypes at different temperatures. However, the historical temperature at which genotypes were reared (26 or 30°C) was not a good predictor of contemporary temperature response. We found increased photosynthetic rates and decreased respiration rates with increasing contemporary temperature, and differences in physiology among genotypes, but found no significant differences in the response of these traits to temperature among genotypes. In species with such broad thermal tolerance, selection experiments on symbionts outside of the host may not yield results sufficient for evolutionary rescue from climate change.

## Graphical abstract

Our work presents the results of an experiment that examines individual variation in symbionts, isolated and grown at different temperatures for hundreds of generations, from an octocoral species. We exposed replicates of different genotypes to increasing temperature and measured their population dynamics and physiology. We found broad thermal tolerance of this symbiont species, but also found significant variation among genotypes, although this was unrelated to the historical temperature at which they were grown.

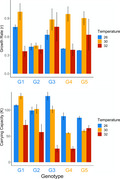

## 1 INTRODUCTION

As anthropogenic changes to the planet increasingly threaten ecosystems, species can respond in four ways (Hughes, 2000, Williams et al., 2008). They can migrate to new habitats that resemble the environmental conditions to which they are adapted (Wallingford et al., 2020). Individuals and populations can acclimate to changing environments via phenotypic plasticity in traits that allow them to persist in the same habitat (Williams et al., 2008). Genetic changes in populations can lead to adaptation to changing environments if driven by natural selection on individuals with heritable traits that confer greater fitness in the new environment (Carlson et al., 2014; Gomulkiewicz & Holt, 1995). Populations that are strongly affected by a changing environment, but unable to respond in any of these ways, are likely to go extinct (Hughes, 2000).

The nature of the response of species to anthropogenic change may depend on the extent of genetic variation in traits among individuals within a species. Ample evidence suggests that ecological responses to environmental changes are influenced by the extent of genetic variation within a population (Bolnick et al., 2011; Hughes et al., 2008; Violle et al., 2012) and that within‐species variation can be as important for determining ecological outcomes as variation among species (Des Roches et al., 2018). Although the benefits of genetic diversity can derive from complementarity, in which some individual genotypes perform better in the presence of other individual genotypes, benefits often arise from increased sampling of individual genotypes that are more tolerant of environmental change (Hughes et al., 2008; Reusch et al., 2005).

Understanding variation in individual responses to environmental shifts may be critical for predicting ecological responses to anthropogenic change (Bolnick et al., 2011; Forsman, 2014). Populations may be better equipped to respond to changes to the environment when the population's niche includes the altered environmental conditions. Often species described as generalists are composed of a population of individual specialists (Bolnick et al., 2003), so individual variation can allow the broader population to fill more niche space. The increase in niche use or different responses to environmental change can allow populations with greater genetic diversity to be more resistant to disturbance and lead to greater stability and increases in ecosystem function (Hughes & Stachowicz, 2004; Schweitzer et al., 2011). The presence of genotypes tolerant to environmental change can allow acclimation of populations to such change. For example, individual genotypes of wild emmer wheat showed variation in their response to temperature and water stress, allowing the broader population to acclimate to changes in temperature and water availability (Li et al., 1999). Additionally, genotype by environment (GxE) interactions can fuel natural selection for genotypes with traits more adapted to a new environment and can result in rapid evolution in response to a changing environment (Hairston et al., 2005; Thompson, 1998). Increasing temperature resulted in the evolution of temperature tolerance in a diatom population because genotypes that were capable of maintaining high growth rates at that temperature became more common in the population (O’Donnell et al., 2018).

Coral reefs around the world are in crisis, due to a number of factors, but chiefly increasing ocean temperatures (Brown, 1997; Glynn & D’Croz, 1990; Hoegh‐Guldberg, 1999). When ocean temperatures exceed a threshold, the mutualism between coral reef species and their dinoflagellate algal symbionts breaks down, resulting in coral bleaching. Given the dependence of hosts on photosynthetically derived carbon from the algae, bleaching often results in the death of the host organism (Eakin et al., 2010, 2019; Glynn & D’Croz, 1990). Given sufficient time, coral reef species and their associated symbionts may acclimate or evolve in response to increasing ocean temperatures, but predictions of severe or total reef loss by 2050, and the consequences for the multitude of species that are associated with reefs, are dire (Heron et al., 2016; Hughes et al., 2018; Oliver et al., 2018; van Hooidonk et al., 2016).

Standing genetic variation among individuals in response to temperature may offer some hope in the face of these worrisome circumstances. The temperature at which bleaching occurs depends largely on the traits of both the host and the algal symbionts (Baird et al., 2009; Quigley et al., 2018). Acclimation to temperature may occur if hosts are able to shuffle or switch symbionts with more temperature‐tolerant strains from either background populations within the host or populations in the ocean (Baker et al., 2004; Berkelmans & van Oppen, 2006; Buddemeier & Fautin, 1993; Jones et al., 2008). Standing variation in traits that confer thermal tolerance may allow for human‐assisted evolution of reef species via artificial selection on symbionts (van Oppen et al., 2015). Both of these mechanisms of acclimation and adaptation via symbionts require standing genetic variation in thermal tolerance. There is some evidence that these mechanisms are possible on some reefs. Measurements of growth rates of symbionts in vitro suggest that there may be sufficient standing variation in thermal tolerance within species in many cases (Bayliss et al., 2019; Díaz‐Almeyda et al., 2017; Grégoire et al., 2017; Pelosi et al., 2021). Symbiont genotypes from historically warmer reefs can allow for higher growth rates of hosts at higher temperatures (Howells et al., 2012). There is also emerging evidence that symbiont evolution in response to increased temperature can reduce bleaching (Chakravarti et al., 2017; Chakravarti & van Oppen, 2018; Zilber‐Rosenberg & Rosenberg, 2008).

Here we quantified the population dynamics and physiology of several genotypes of a single species of algal symbiont that resulted from a long‐term selection experiment at different temperatures. *Breviolum antillogorgium* is the dominant symbiont found in octocoral hosts from the genus *Antillogorgia*. Symbionts are transmitted horizontally and acquired from the environment. Our selection experiment and following in vitro experiments are likely to mimic conditions in the environment, rather than in hospite, but we measured traits likely to affect the strength of the mutualism with the host that may ultimately determine which symbionts are able to successfully colonize hosts. We used the genotypes resulting from the selection experiment to quantify changes in traits in response to increasing temperature (acclimation) and effects of historical temperature of the selection experiment (adaptation), and to identify standing genetic variation in thermal tolerance that could allow for further acclimation or adaptation in the future. In a previous study, we found broad thermal tolerance in growth rate and host survival among genotypes of *Breviolum antillogorgium* up to 30°C (Pelosi et al., 2021). Here we exposed these same genotypes to temperatures up to 32°C while also measuring additional traits, including growth rate, maximum sustainable population size, photosynthetic rate, and respiration rate. Algal symbionts trade photosynthetically derived carbon in exchange for nitrogen waste and other nutrients from their hosts. Respiration and photosynthesis affect the amount of carbon available for trade with the host, and thus may affect the strength of the mutualism. Symbiont growth rate and population dynamics may have a non‐linear effect on benefits to the host. At low symbiont densities inside hosts, rapid growth or high carrying capacity may allow the symbiont population to grow quickly and provide resources, but at high symbiont densities, rapid growth or overcrowding could lead to less net benefit per cell (Cunning & Baker, 2014).

## 2 METHODS

The genotypes used in this study were the same as those used by Pelosi et al. (2021). Briefly, we collected octocoral colonies in the genus *Antillogorgia* from Elbow Reef and Pickles Reef in the Florida Keys in September 2016 (11–18 m depth), homogenized the octocoral tissue samples, and collected the symbionts by centrifugation. The resultant heterogenous slurry of symbiont strains was added to flasks with 30 ml f/2 media (Guillard & Ryther, 1962), and replicate flasks (*n* = 3) from each colony were immediately placed at and maintained at 26 and 30°C as a selection experiment for genotypes capable of survival and growth at the respective temperatures. After a year at these temperatures, molecular analysis confirmed that single genotypes dominated most flasks (Pelosi et al., 2021). As *Breviolum antillogorgium* appears to be specialized for octocorals within the genus *Antillogorgia*¸ we focused our study on this symbiont species and recovered three *B. antillogorgium* genotypes from the 26°C treatment (G1, G2, G3) and two *B. antillogorgium* genotypes from the 30°C treatment (G4, G5). Following isolation, these five genotypes were kept in monocultures at their respective temperatures for an additional 5 years (~650 to 700 generations), allowing for the potential for accumulation of mutations in each genotype. These five genotypes were used in the present study. We confirmed the presence of a single algal genotype in each culture using five microsatellite loci (Andras et al., 2009; Pettay & Lajeunesse, 2007; Santos et al., 2003), but cultures were not axenic with respect to bacteria, archaea, and fungi, which we did not quantify. All cultures were maintained in identical growth chambers and transferred to fresh f/2 media monthly. Further details of collection and genotyping are available in Pelosi et al. (2021).

In March 2021, we initiated a laboratory experiment to measure population dynamics and physiology of each genotype at three different temperatures. We used the stock cultures of each genotype to initiate 15 new replicate 50‐ml cultures at an initial density of 10,000 cells/ml. Five of these cultures of each genotype were maintained in a growth chamber set at 26°C (actual mean temperature ±SD determined by HOBO Data Logger: 25.5°C ± 0.51). Another five cultures of each genotype were grown in identical growth chambers set at 30°C (30.1° ± 0.28) and 32°C (31.6° ± 0.23). Lights were set on a 12:12 day:night cycle, with average day illumination of 4244 Lux (approximately 59 µmole m^−2^ s^−1^ based on a conversion of 1 lux = 0.014 µmole m^−2^ s^−1^). Mean temperature data from Elbow Reef from 2005–2015 indicate that in some years, mean temperatures at Elbow Reef can exceed 30°C for months, but in other years, mean temperatures do not reach 30°C; however, the warmest years in that time period are the most recent (Pelosi et al., 2021).

Every 3 days, we removed 50 μl from each culture and performed four replicate hemacytometer counts and used the mean as an estimate of cell density. We estimated densities over time for 34 days, by which time, all cultures had peaked in density and were in steady‐state growth. Due to a scheduling error, the cultures grown at 30°C were not counted on day 19. We used the time series data up to the time of maximum density in each culture to estimate per‐capita growth rate (r) and carrying capacity (K) using the “growthrates” package (Petzoldt, 2019) in R v. 4.0.2 (R Core Team, 2020).

On Day 27 of the experiment, when all cultures were in steady‐state growth, we removed 2 ml from each culture grown at 26°C and used these samples to estimate rates of photosynthesis and respiration at each temperature. Replicate samples were placed in randomly assigned wells in a microrespirometry plate that quantified changes in oxygen concentrations over time (Loligo Systems, Viborg, Denmark) in the 26°C growth chamber. We also placed sterile f/2 samples in two wells in each plate to account for any background changes in oxygen concentration. Samples were dark‐adapted for 10 min before measuring oxygen levels in each well every 15 s for 10 min. Following this, we turned the lights on in the growth chambers, allowed samples two minutes to acclimate, and then again measured oxygen levels every 15 s for 10 min. The microrespirometry plates were then moved to the 30°C growth chamber, and later the 32°C growth chamber, and allowed to acclimate for 15 min in each growth chamber before measurements were taken. We estimated respiration as the slope of a linear fit to declining oxygen levels over time in the dark, subtracting any background changes in oxygen. Similarly, we estimated net photosynthesis as the slope of a linear fit to increasing oxygen levels, accounting for background changes in oxygen in the light. We estimated gross productivity by adding the absolute value of respiration in each culture to net photosynthesis. We standardized respiration, net photosynthesis, and gross photosynthesis by the number of cells in each well, determined by replicate hemacytometer counts, as above. We used the mean of the two replicate measurements of respiration and photosynthesis for each culture as the estimate for each culture.

At the end of the experiment, samples of each replicate were preserved in 95% ethanol. DNA was extracted and amplified following the methods in Pelosi et al. (2021) to verify symbiont genotype at the end of the experiment. We used Analysis of Variance (ANOVA) to determine the fixed effects of historical temperature, contemporary temperature, and their interaction on maximum growth rate (r), carrying capacity (K), respiration, gross photosynthesis, and net photosynthesis in separate tests. Genotypes were nested within historical temperature. All data were visually inspected for normality and heteroscedasticity using Q–Q plots and plots of residuals against fitted values. All data met the assumptions of ANOVA, except for r, which was log‐transformed to meet assumptions. We performed model selection using Akaike's Information Criterion (AIC). When necessary, we used Tukey's post‐hoc tests to compare individual genotypes to each other. All analyses were conducted in the R Statistical Computing Platform (v. 4.0.2).

## 3 RESULTS

At the end of the experiment, genetic analyses indicated that each culture contained only the genotype we expected, except for cultures of G3, which contained both G1 and G3. The extent of contamination suggests that the stock culture of G3 likely contained both G1 and G3. We do not know the relative abundances of genotypes in these cultures, so observed results may be driven largely by either G1 or G3, or a combination of the two. Here we present results without genotype G3 but include results of analyses including G3 in the supplemental material.

**FIGURE 1:**
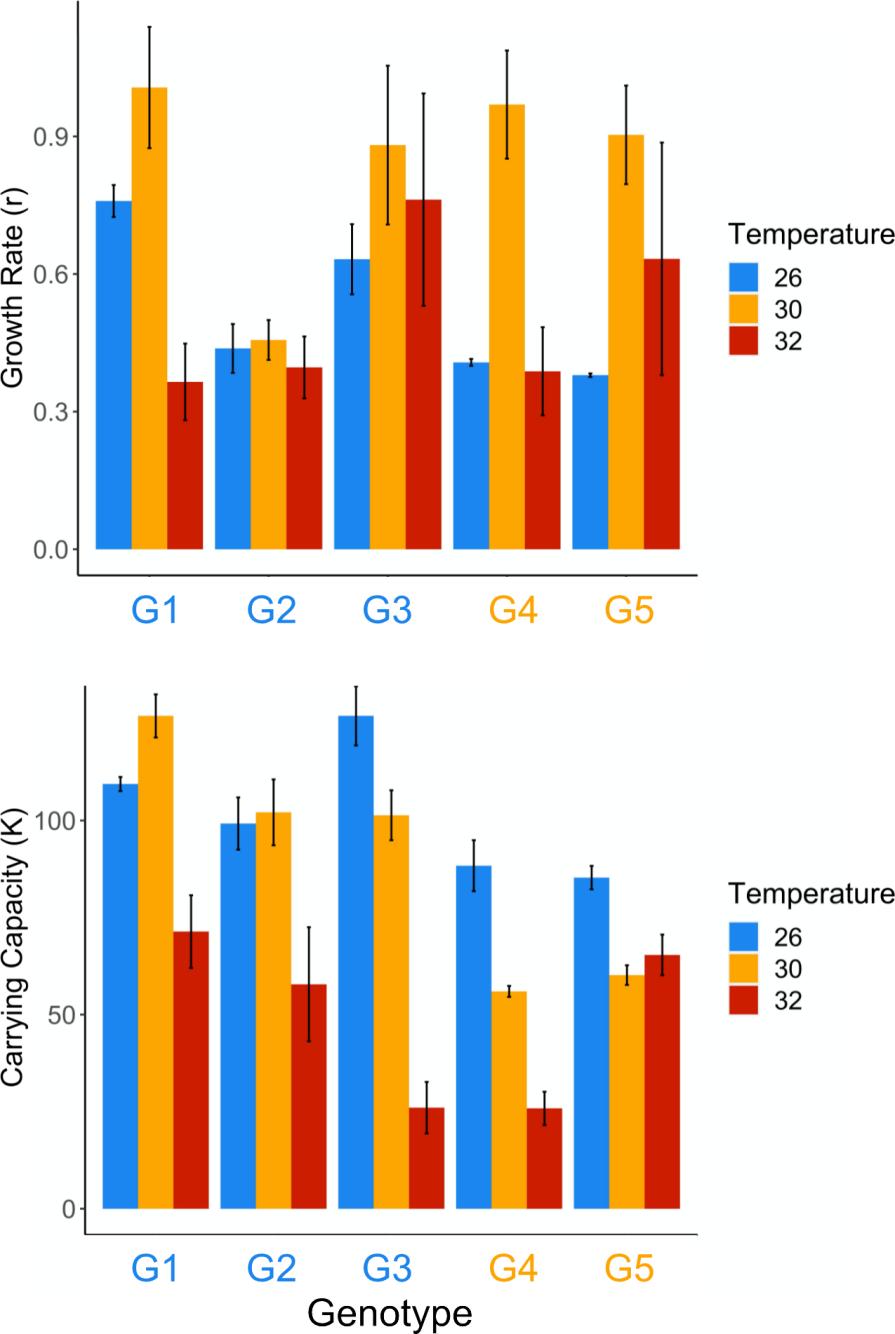
Mean (±SE) growth rate (a) and carrying capacity (b) of four genotypes of *Breviolum antillogorgium* grown at three temperatures. Genotypes in blue (G1–G2) were isolated and grown at 26°C and genotypes in orange (G4–G5) were isolated and grown at 30°C

All genotypes had positive growth rates at each temperature. However, different genotypes had different growth rate responses to increasing temperature (Temperature * Genotype: *F*_6,48_ = 3.55, *p* = .005). Although every genotype experienced the highest mean growth rate at 30°C, the extent to which growth rates dropped at 32°C varied among genotypes. For example, G1 and G4 showed a steep decline in growth rate at the highest temperature, but G2 showed little decline (Figure 1a). Similarly, the extent to which growth rate increased between 26°C and 30°C varied among genotypes, with sharp increases observed for G1, G4, and G5, but little difference observed for G2 (Figure 1a). Although carrying capacity tended to be highest at 26°C, we observed more variable responses among genotypes in the response of carrying capacity to temperature (Temperature * Genotype: *F*_6,48_ = 5.53, *p* < .001). Genotype G4 showed a steady decline in K with increasing temperature (Figure 1b). G1 showed a peak in K at 30°C and G2 showed a decrease in K at 32°C, but G5 showed relatively little variation with temperature (Figure 1b). Historical temperature was not a part of either best‐fit model (ΔAIC = 15.5 for r; ΔAIC = 16.2 for K).

**FIGURE 2:**
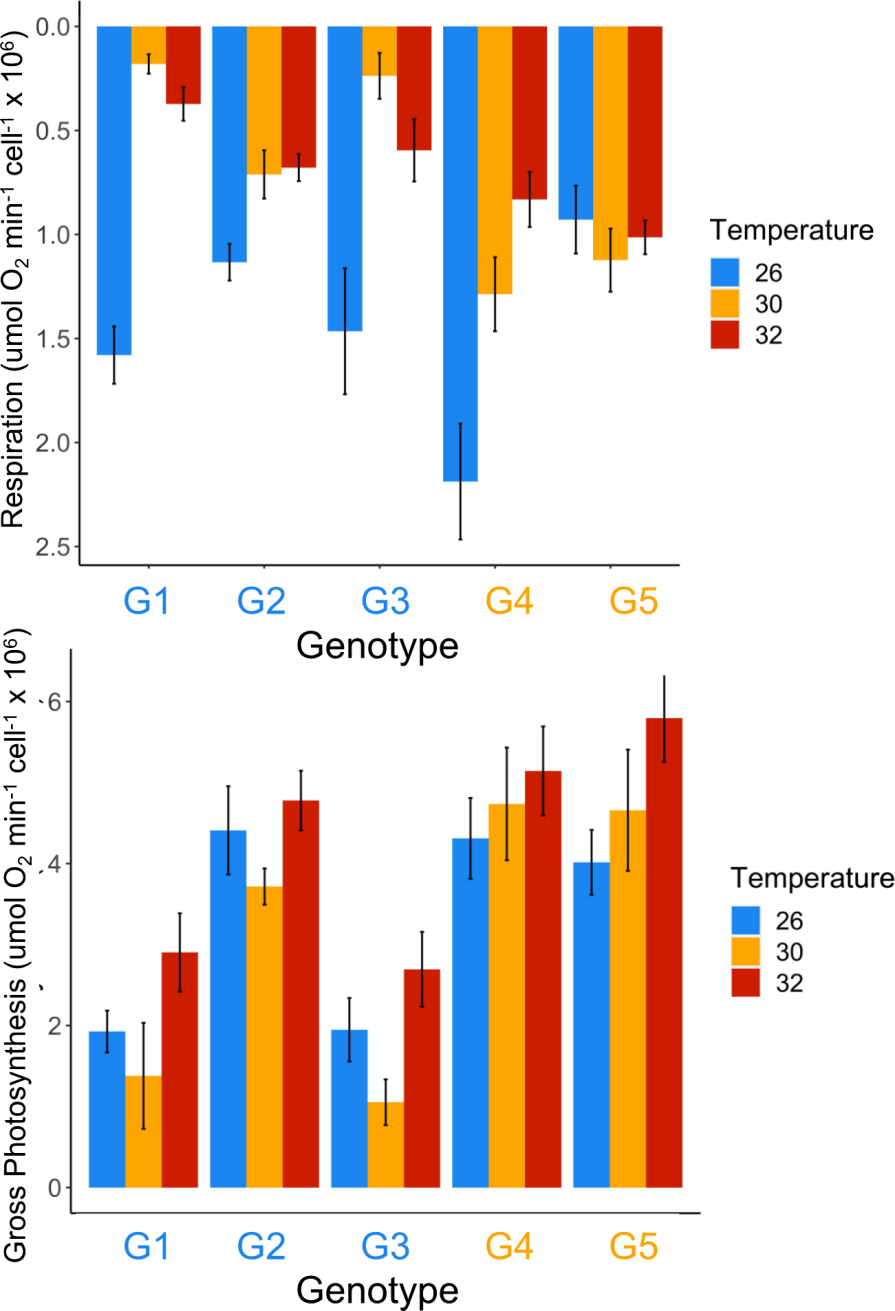
Mean (±SE) respiration rate (a) and gross photosynthetic rate (b) of four genotypes of *Breviolum antillogorgium* grown at three temperatures. Rates were standardized by cell density. Genotypes in blue (G1–G2) were isolated and grown at 26°C and genotypes in orange (G4–G5) were isolated and grown at 30°C

**FIGURE 3:**
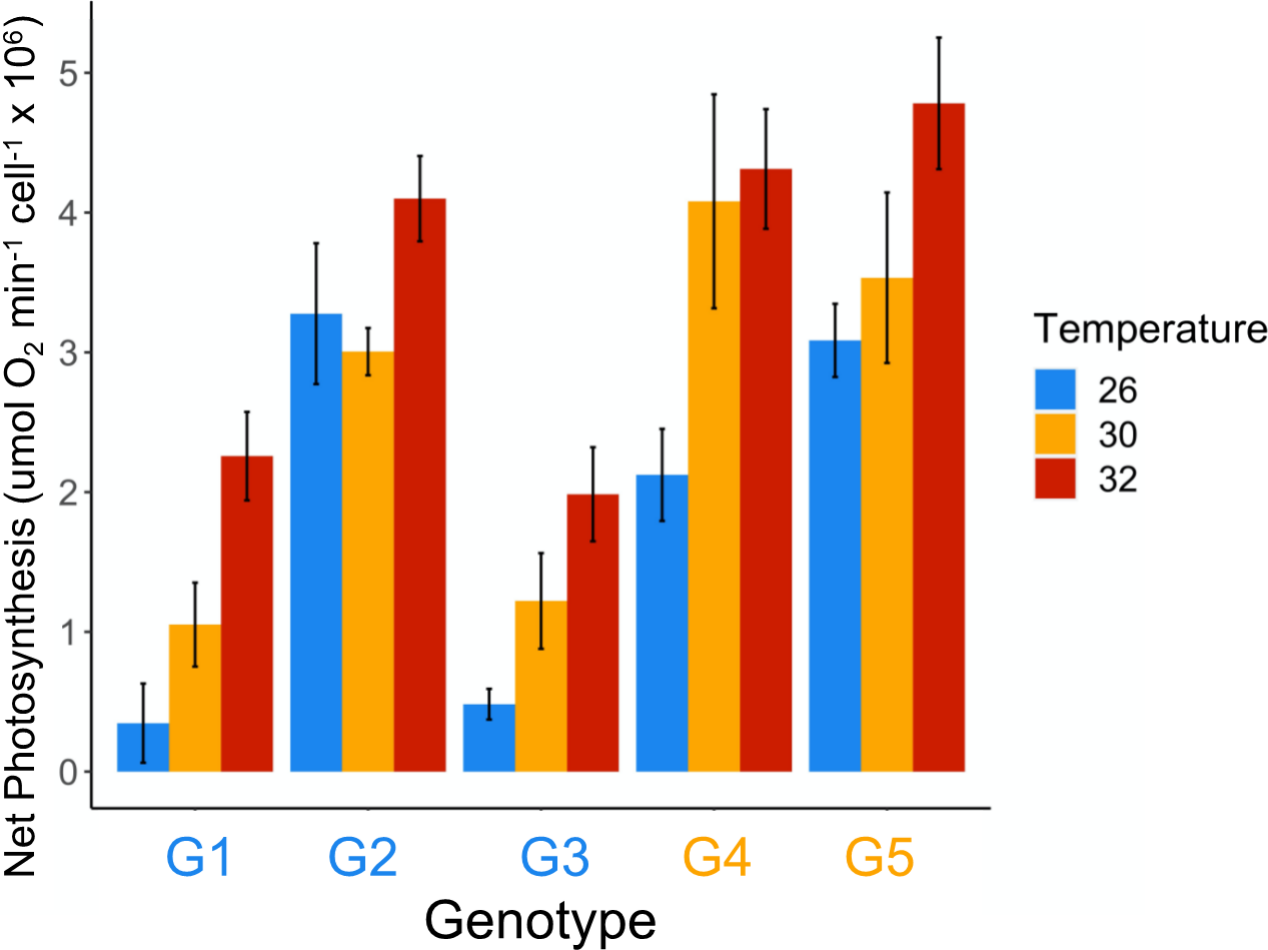
Mean (±SE) net photosynthetic rate of four genotypes of *Breviolum antillogorgium* grown at three temperatures, standardized by cell density. Genotypes in blue (G1–G2) were isolated and grown at 26°C and genotypes in orange (G4–G5) were isolated and grown at 30°C

All genotypes demonstrated the ability to acclimate their physiology to each temperature to some extent, but these responses did not reveal a strong effect of historical temperature (ΔAIC = 13.8 for respiration; ΔAIC = 11.8 for gross photosynthesis; ΔAIC = 14.4 for net photosynthesis). Genotypes had different respiration responses at different temperatures (Temperature * Genotype: *F*_6,48_ = 8.38, *p* < .001). One genotype (G5) showed little variation in respiration across temperatures. The remaining three genotypes showed decreases in respiration at higher temperatures (30 and 32°C) relative to 26°C, but the magnitude of the decrease varied among genotypes (Figure 2a). Temperature also had a significant effect on gross photosynthesis (*F*_2,48_ = 5.03, *p* = .010), and genotypes differed in gross photosynthetic rate (*F*_3,48_ = 28.5, *p* < .001), but, in contrast to the respiration results, there was no significant difference among genotypes in response to increasing temperature (Temperature * Genotype: *F*_6,48_ = 0.624, *p* = .710). Gross photosynthetic rate increased with increasing temperature, and genotypes G2, G4, and G5 tended to have higher photosynthetic rates than G1 (Figure 2b). Patterns of net photosynthetic rate were similar to those for gross photosynthesis; temperature (*F*_2,48_ = 15.1, *p* < .001) and genotype (*F*_3,48_ = 23.5, *p* < .001) had a significant effect on net photosynthesis, but again, there was no significant interaction between temperature and genotype (*F*_6,48_ = 1.32, *p* = .267, Figure 3).

## 4 DISCUSSION

Our results demonstrate broad thermal tolerance in symbionts isolated from *Antillogorgia* octocorals, with positive growth at temperatures up to 32°C, which is beyond the bleaching threshold observed in many coral reef species (Berkelmans, 2002). We also observed the highest photosynthetic rates and lowest respiration rates at higher temperatures, suggesting that the potential benefits these symbionts can provide to their mutualist hosts are considerable at temperatures that appear to be stressful to many reef hosts. Population dynamics and physiology were largely dependent on genotype, suggesting that individual variation among symbionts may be important for the strength of mutualism. The lack of difference between historical temperatures suggests that the observed responses are more likely the result of acclimation to different temperatures, rather than evolution in response to historical temperatures.

The increased photosynthetic rates we observed with increasing temperature may be explained by increased enzyme activity at higher temperatures, which can be especially important for acclimation to temperature changes (Iglesias‐Prieto et al., 1992). An increase in net photosynthesis with increasing temperature suggests that symbionts have more carbon available to provide to the host and may be beneficial across this range of temperatures. Increased respiration rates are often a sign of physiological stress for many organisms, but we generally observed decreased respiration rates with increasing temperature, further indicating broad thermal tolerance. Notably, we measured respiration and photosynthesis at steady‐state population growth, so these measures may not reflect physiology during exponential population growth. However, as hosts typically regulate symbiont cell densities, physiology during steady‐state growth may best reflects the strength of mutualism with the host. Although the response of respiration to temperature varied among genotypes, photosynthetic responses to temperature did not. Across species of Symbiodinaceae, respiration and photosynthesis may become decoupled at different temperatures, with respiration often more sensitive to temperature than photosynthesis (Pierangelini et al., 2020). These responses are species‐specific, with strong coupling in some species and strong decoupling in other species (Pierangelini et al., 2020). If similar mechanisms occur within species, this may help to explain the different responses of respiration and photosynthesis among genotypes in this study.

Although we observed increased growth rates between 26 and 30°C, this is in contrast to Pelosi et al. (2021), where decreased growth rates were observed at 30°C in the same genotypes. We generally did not observe decreased growth rates until 32°C. Potentially, this difference could be driven by the rise of beneficial mutations over the hundreds of generations between studies. An alternative explanation is that these two studies were conducted in different laboratories (University at Buffalo vs. California State University, Northridge). Although the mean and variance in temperatures were similar between growth chambers in different laboratories, the light levels in this study were ~50% to 58% of the intensity of those in the previous study (~60 µmols m^−2^ s^−1^ here, vs. 103.8 to 120.6 µmols m^−2^ s^−1^ in Pelosi et al., 2021). The seawater in both labs was made from Instant Ocean, but the University at Buffalo water comes from the Aquarium of Niagara, in tanks with animals and may have higher nitrogen content. For many photosynthetic organisms, the optimal growth temperature depends on the light and nutrient environment (Edwards et al., 2016), which may explain the discrepancy between studies. This also highlights the potential importance of light and nutrient levels in conducting selection experiments in the laboratory and how they may change in natural environments or inside host tissues (Grottoli et al., 2021).

This experiment demonstrates significant differences among algal genotypes, but these differences were observed in vitro. These algae spend a portion of their life cycle outside the host, and in vitro conditions might better mimic this part of the life cycle, although nutrient levels are certainly initially higher in f/2 media than in tropical oceans (Bayliss et al., 2019). The portion of their life cycle of most interest to those studying mutualisms in coral reef ecology is that spent *in* hospite. Whether these in vitro differences translate to effects on holobiont responses to temperature remains to be seen. Individual differences in traits outside of the host may be important if hosts regularly switch symbionts and incorporate new symbionts with different traits from the environment. Because newly settled polyps take up symbionts from the environment, the potential for such switching occurs at least every generation, although newly settled polyps reduce uptake after 5 months (McIlroy & Coffroth, 2017), suggesting limited exchange in adults of this host species. The genotypes in this experiment affected symbiont density at different temperatures in young polyps but had no effect on polyp survival at increased temperatures (Pelosi et al., 2021). Hosts are likely to regulate symbiont growth rates, maximum density, and physiology, but the magnitude of this effect relative to the differences among genotypes is unknown.

Our results suggest that each genotype has a different optimum temperature for growth, respiration, and photosynthesis. However, these optima measured *in vitro* may not be the same as what is optimal for the holobiont. When considering a single symbiont species, long‐term growth rate of a genotype would be a good proxy for fitness, and covariances between traits and fitness should be good estimates of selection on those traits. However, several studies indicate that symbiont physiologies vary between in vitro and in hospite (Bellantuono et al., 2019; Bhagooli & Hidaka, 2003; Ravelo & Conaco, 2018). Thus, when considering evolution in a community context (terHorst et al., 2018), where fitness of the host and associated microbes are tightly linked, selection on the holobiont might not be easily predicted from studies in monocultures. For example, increased symbiont growth rates that are indicative of high fitness in vitro or metabolic demand in response to temperature stress might result in increased demand for resources from the host and subsequent breakdown of the mutualism (Rädecker et al., 2021). Although nitrogen is abundant in vitro, at least initially, it is likely to be more limiting in hospite, with increased nitrogen often destabilizing the mutualism (Morris et al., 2019; Rädecker et al., 2015). The broad availability of nutrients when conducting selection experiments in vitro may obscure trade‐offs with other algal traits, trade‐offs with host traits, or trade‐offs with other traits only observed in the context of the holobiont (Chan et al., 2021). Selection experiments on symbionts outside of the host may not yield the evolutionary rescue necessary to adapt to climate change, but rather may require selection experiments on the holobiont.

Conservation‐minded assisted evolution for coral reef organisms proposes that selection experiments in the laboratory could yield temperature‐tolerant symbiont genotypes that could be later used to seed reefs experiencing temperature stress (van Oppen et al., 2015). Selection experiments could be conducted on heterogenous cultures composed of standing genetic variation found within and among hosts on reefs (Chan et al., 2021; Pelosi et al., 2021). The genotypes used in this experiment are the result of a long‐term selection experiment. Heterogenous cultures of algae were allowed to grow at both 26 and 30°C, and the isolated genotypes in this study were unique genotypes that were able to grow well at those temperatures (Pelosi et al., 2021). Additionally, cultures continued to grow at these temperatures for hundreds of generations prior to this experiment and any beneficial mutations would have potentially been subject to positive selection. Nevertheless, we did not observe any obvious effects of historical temperature environment on population dynamics or physiology.

Although our ability to detect an effect of historical temperature was limited to only two genotypes from each temperature, the patterns we observed suggest the opposite of what we would hope for from a successful selection experiment. Genotypes from different evolutionary histories tended to be more similar to each other than genotypes from the same evolutionary history. This suggests that the genotypes recovered from our different selection environments may more likely be the result of genetic drift and random chance than natural selection, or that selection is acting more strongly on traits unrelated to temperature tolerance, such as nutrient uptake rate, that may cause trade‐offs with temperature tolerance. As species on coral reefs have repeatedly been exposed to high temperatures in recent decades (Heron et al., 2016; Hughes et al., 2018; Oliver et al., 2018; van Hooidonk et al., 2016), it is possible that these genotypes are already the result of winnowing of non‐tolerant genotypes and adaptation to temperature in the ocean, making it difficult to artificially impose further selection. For species such as this that exhibit broad thermal tolerance and are able to acclimate to temperature, selection experiments may prove more difficult. Whether temperature tolerance results from past adaptation or contemporary acclimatization, hosts that harbor these thermally tolerant symbionts may have increased resilience to changes in ocean temperatures.

## AUTHOR CONTRIBUTIONS

Casey P. terHorst: Conceptualization (equal); Data curation (lead); Formal analysis (lead); Funding acquisition (equal); Investigation (equal); Methodology (equal); Project administration (equal); Resources (equal); Validation (supporting); Writing—original draft (lead); Writing—review & editing (equal). Mary‐Alice Coffroth: Conceptualization (equal); Data curation (supporting); Formal analysis (supporting); Funding acquisition (equal); Investigation (equal); Methodology (equal); Project administration (equal); Resources (equal); Validation (lead); Writing—original draft (supporting); Writing—review & editing (equal).

## CONFLICT OF INTEREST

The authors declare no competing interests.

## Supporting information

Fig S1‐S3

## Data Availability

Data from this study have been uploaded to the Biological and Chemical Oceanography Data Management Office and are publicly available (https://doi.org/10.26008/1912/bco‐dmo.874587.1).

## References

[ece39000-bib-0001] Andras, J. P. , Kirk, N. L. , Coffroth, M. A. , & Harvell, C. D. (2009). Isolation and characterization of microsatellite loci in Symbiodinium B1/B184, the dinoflagellate symbiont of the Caribbean sea fan coral, Gorgonia ventalina. Molecular Ecology Resources, 9, 989–993. 21564815 10.1111/j.1755-0998.2009.02549.x

[ece39000-bib-0002] Baird, A. H. , Bhagooli, R. , Ralph, P. J. , & Takahashi, S. (2009). Coral bleaching: The role of the host. Trends in Ecology & Evolution, 24, 16–20. 10.1016/j.tree.2008.09.005 19022522

[ece39000-bib-0003] Baker, A. C. , Starger, C. J. , McClanahan, T. R. , & Glynn, P. W. (2004). Corals’ adaptive response to climate change. Nature, 430, 741. 10.1038/430741a 15306799

[ece39000-bib-0004] Bayliss, S. L. J. , Scott, Z. R. , Coffroth, M. A. , & terHorst, C. P. (2019). Genetic variation in Breviolum antillogorgium, a coral reef symbiont, in response to temperature and nutrients. Ecology and Evolution, 9, 2803–2813. 30891218 10.1002/ece3.4959 PMC6406013

[ece39000-bib-0005] Bellantuono, A. J. , Dougan, K. E. , Granados‐Cifuentes, C. , & Rodriguez‐Lanetty, M. (2019). Free‐living and symbiotic lifestyles of a thermotolerant coral endosymbiont display profoundly distinct transcriptomes under both stable and heat stress conditions. Molecular Ecology, 28, 5265–5281. 10.1111/mec.15300 31693775

[ece39000-bib-0006] Berkelmans, R. (2002). Time‐integrated thermal bleaching thresholds of reefs and their variation on the Great Barrier Reef. Marine Ecology Progress Series, 229, 73–82. 10.3354/meps229073

[ece39000-bib-0007] Berkelmans, R. , & van Oppen, M. J. H. (2006). The role of zooxanthellae in the thermal tolerance of corals: A “nugget of hope” for coral reefs in an era of climate change. Proceedings of the Royal Society B: Biological Sciences, 273, 2305–2312. 10.1098/rspb.2006.3567 PMC1636081 16928632

[ece39000-bib-0008] Bhagooli, R. , & Hidaka, M. (2003). Comparison of stress susceptibility of in hospite and isolated zooxanthellae among five coral species. Journal of Experimental Marine Biology and Ecology, 291, 181–197. 10.1016/S0022-0981(03)00121-7

[ece39000-bib-0009] Bolnick, D. I. , Amarasekare, P. , Araujo, M. S. , Buerger, R. , Levine, J. M. , Novak, M. , Rudolf, V. H. W. , Schreiber, S. J. , Urban, M. C. , & Vasseur, D. A. (2011). Why intraspecific trait variation matters in community ecology. Trends in Ecology & Evolution, 26, 183–192. 10.1016/j.tree.2011.01.009 21367482 PMC3088364

[ece39000-bib-0010] Bolnick, D. , Svanbäck, R. , Fordyce, J. A. , Yang, L. H. , Davis, J. M. , Hulsey, C. D. , & Forister, M. L. (2003). The ecology of individuals: Incidence and implications of individual specialization. American Naturalist, 161, 1–28. 10.1086/343878 12650459

[ece39000-bib-0011] Brown, B. E. (1997). Coral bleaching: Causes and consequences. Coral Reefs, 16, S129–S138. 10.1007/s003380050249

[ece39000-bib-0012] Buddemeier, R. , & Fautin, D. (1993). Coral bleaching as an adaptive mechanism: A testable hypothesis. BioScience, 43, 320–326. 10.2307/1312064

[ece39000-bib-0013] Carlson, S. M. , Cunningham, C. J. , & Westley, P. A. H. (2014). Evolutionary rescue in a changing world. Trends in Ecology & Evolution, 29, 521–530. 10.1016/j.tree.2014.06.005 25038023

[ece39000-bib-0014] Chakravarti, L. J. , Beltran, V. H. , & van Oppen, M. J. H. (2017). Rapid thermal adaptation in photosymbionts of reef‐building corals. Global Change Biology, 23, 4675–4688. 10.1111/gcb.13702 28447372

[ece39000-bib-0015] Chakravarti, L. J. , & van Oppen, M. J. H. (2018). Experimental evolution in coral photosymbionts as a tool to increase thermal tolerance. Frontiers in Marine Science, 5, 227. 10.3389/fmars.2018.00227

[ece39000-bib-0016] Chan, W. Y. , Oakeshott, J. G. , Buerger, P. , Edwards, O. R. , & van Oppen, M. J. H. (2021). Adaptive responses of free‐living and symbiotic microalgae to simulated future ocean conditions. Global Change Biology, 27, 1737–1754. 10.1111/gcb.15546 33547698

[ece39000-bib-0017] Cunning, R. , & Baker, A. C. (2014). Not just who, but how many: The importance of partner abundance in reef coral symbioses. Frontiers in Microbiology, 5, 400. 25136339 10.3389/fmicb.2014.00400 PMC4120693

[ece39000-bib-0018] Des Roches, S. , Post, D. M. , Turley, N. E. , Bailey, J. K. , Hendry, A. P. , Kinnison, M. T. , Schweitzer, J. A. , & Palkovacs, E. P. (2018). The ecological importance of intraspecific variation. Nature Ecology & Evolution, 2, 57–64. 10.1038/s41559-017-0402-5 29203921

[ece39000-bib-0019] Díaz‐Almeyda, E. M. , Prada, C. , Ohdera, A. H. , Moran, H. , Civitello, D. J. , Iglesias‐Prieto, R. , Carlo, T. A. , LaJeunesse, T. C. , & Medina, M. (2017). Intraspecific and interspecific variation in thermotolerance and photoacclimation in Symbiodinium dinoflagellates. Proceedings of the Royal Society B: Biological Sciences, 284, 20171767. 10.1098/rspb.2017.1767 PMC5740277 29212723

[ece39000-bib-0020] Eakin, C. M. , Morgan, J. A. , Heron, S. F. , Smith, T. B. , Liu, G. , Alvarez‐Filip, L. , Baca, B. , Bartels, E. , Bastidas, C. , Bouchon, C. , Brandt, M. , Bruckner, A. W. , Bunkley‐Williams, L. , Cameron, A. , Causey, B. D. , Chiappone, M. , Christensen, T. R. L. , Crabbe, M. J. C. , Day, O. , … Yusuf, Y. (2010). Caribbean corals in crisis: Record thermal stress, bleaching, and mortality in 2005. PLoS One, 5, e13969. Public Library of Science. 21125021 10.1371/journal.pone.0013969 PMC2981599

[ece39000-bib-0021] Eakin, C. M. , Sweatman, H. P. A. , & Brainard, R. E. (2019). The 2014–2017 global‐scale coral bleaching event: Insights and impacts. Coral Reefs, 38, 539–545. 10.1007/s00338-019-01844-2

[ece39000-bib-0022] Edwards, K. F. , Thomas, M. K. , Klausmeier, C. A. , & Litchman, E. (2016). Phytoplankton growth and the interaction of light and temperature: A synthesis at the species and community level. Limnology and Oceanography, 61, 1232–1244. 10.1002/lno.10282

[ece39000-bib-0023] Forsman, A. (2014). Effects of genotypic and phenotypic variation on establishment are important for conservation, invasion, and infection biology. Proceedings of the National Academy of Sciences, 111, 302–307. 10.1073/pnas.1317745111 PMC3890895 24367109

[ece39000-bib-0024] Glynn, P. W. , & D’Croz, L. (1990). Experimental evidence for high temperature stress as the cause of El Niño‐coincident coral mortality. Coral Reefs, 8, 181–191.

[ece39000-bib-0025] Gomulkiewicz, R. , & Holt, R. D. (1995). When does evolution by natural selection prevent extinction? Evolution, 49(1), 201–207. [Society for the Study of Evolution, Wiley]. 28593677 10.1111/j.1558-5646.1995.tb05971.x

[ece39000-bib-0026] Grégoire, V. , Schmacka, F. , Coffroth, M. A. , & Karsten, U. (2017). Photophysiological and thermal tolerance of various genotypes of the coral endosymbiont Symbiodinium sp. (Dinophyceae). Journal of Applied Phycology, 29. 10.1007/s10811-017-1127-1

[ece39000-bib-0027] Grottoli, A. G. , Toonen, R. J. , van Woesik, R. , Vega Thurber, R. , Warner, M. E. , McLachlan, R. H. , Price, J. T. , Bahr, K. D. , Baums, I. B. , Castillo, K. D. , Coffroth, M. A. , Cunning, R. , Dobson, K. L. , Donahue, M. J. , Hench, J. L. , Iglesias‐Prieto, R. , Kemp, D. W. , Kenkel, C. D. , Kline, D. I. , … Wu, H. C. (2021). Increasing comparability among coral bleaching experiments. Ecological Applications, 31, e02262. 10.1002/eap.2262 33222325 PMC8243963

[ece39000-bib-0028] Guillard, R. , & Ryther, J. (1962). Studies of marine planktonic diatoms. 1. Cyclotella Nana Hustedt, and Detonula Confervacea (cleve) Gran. Canadian Journal of Microbiology, 8, 229–239. 13902807 10.1139/m62-029

[ece39000-bib-0029] Hairston, N. G. , Ellner, S. P. , Geber, M. A. , Yoshida, T. , & Fox, J. A. (2005). Rapid evolution and the convergence of ecological and evolutionary time. Ecology Letters, 8, 1114–1127. 10.1111/j.1461-0248.2005.00812.x

[ece39000-bib-0030] Heron, S. F. , Maynard, J. A. , van Hooidonk, R. , & Eakin, C. M. (2016). Warming trends and bleaching stress of the world’s coral reefs 1985–2012. Scientific Reports, 6, 38402. 10.1038/srep38402 27922080 PMC5138844

[ece39000-bib-0031] Hoegh‐Guldberg, O. (1999). Climate change, coral bleaching and the future of the world’s coral reefs. Marine & Freshwater Research, 50, 839–866. 10.1071/MF99078

[ece39000-bib-0032] Howells, E. J. , Beltran, V. H. , Larsen, N. W. , Bay, L. K. , Willis, B. L. , & van Oppen, M. J. H. (2012). Coral thermal tolerance shaped by local adaptation of photosymbionts. Nature Climate Change, 2, 116–120. 10.1038/nclimate1330

[ece39000-bib-0033] Hughes, A. R. , Inouye, B. D. , Johnson, M. T. J. , Underwood, N. , & Vellend, M. (2008). Ecological consequences of genetic diversity. Ecology Letters, 11, 609–623. 10.1111/j.1461-0248.2008.01179.x 18400018

[ece39000-bib-0034] Hughes, A. R. , & Stachowicz, J. J. (2004). Genetic diversity enhances the resistance of a seagrass ecosystem to disturbance. Proceedings of the National Academy of Sciences of the United States of America, 101, 8998–9002. 10.1073/pnas.0402642101 15184681 PMC428461

[ece39000-bib-0035] Hughes, L. (2000). Biological consequences of global warming: Is the signal already apparent? Trends in Ecology & Evolution, 15, 56–61. 10.1016/S0169-5347(99)01764-4 10652556

[ece39000-bib-0036] Hughes, T. P. , Anderson, K. D. , Connolly, S. R. , Heron, S. F. , Kerry, J. T. , Lough, J. M. , Baird, A. H. , Baum, J. K. , Berumen, M. L. , Bridge, T. C. , Claar, D. C. , Eakin, C. M. , Gilmour, J. P. , Graham, N. A. J. , Harrison, H. , Hobbs, J.‐P.‐A. , Hoey, A. S. , Hoogenboom, M. , Lowe, R. J. , … Wilson, S. K. (2018). Spatial and temporal patterns of mass bleaching of corals in the Anthropocene. Science, 359, 80–83. 10.1126/science.aan8048 29302011

[ece39000-bib-0037] Iglesias‐Prieto, R. , Matta, J. L. , Robins, W. A. , & Trench, R. K. (1992). Photosynthetic response to elevated temperature in the symbiotic dinoflagellate Symbiodinium microadriaticum in culture. Proceedings of the National Academy of Sciences, 89(21), 10302–10305. 10.1073/pnas.89.21.10302 PMC50326 11607337

[ece39000-bib-0038] Jones, A. M. , Berkelmans, R. , van Oppen, M. J. H. , Mieog, J. C. , & Sinclair, W. (2008). A community change in the algal endosymbionts of a scleractinian coral following a natural bleaching event: Field evidence of acclimatization. Proceedings of the Royal Society B: Biological Sciences, 275, 1359–1365. 10.1098/rspb.2008.0069 PMC2367621 18348962

[ece39000-bib-0039] Li, Y. C. , Fahima, T. , Beiles, A. , Korol, A. B. , & Nevo, E. (1999). Microclimatic stress and adaptive DNA differentiation in wild emmer wheat, Triticum dicoccoides. TAG. Theoretical and Applied Genetics, 98, 873–883. 10.1007/s001220051146

[ece39000-bib-0040] McIlroy, S. E. , & Coffroth, M. A. (2017). Coral ontogeny affects early symbiont acquisition in laboratory‐reared recruits. Coral Reefs, 36, 927–932. 10.1007/s00338-017-1584-7

[ece39000-bib-0041] Morris, L. A. , Voolstra, C. R. , Quigley, K. M. , Bourne, D. G. , & Bay, L. K. (2019). Nutrient availability and metabolism affect the stability of coral‐symbiodiniaceae symbioses. Trends in Microbiology, 27, 678–689. 10.1016/j.tim.2019.03.004 30987816

[ece39000-bib-0042] O’Donnell, D. R. , Hamman, C. R. , Johnson, E. C. , Kremer, C. T. , Klausmeier, C. A. , & Litchman, E. (2018). Rapid thermal adaptation in a marine diatom reveals constraints and trade‐offs. Global Change Biology, 24, 4554–4565. 10.1111/gcb.14360 29940071

[ece39000-bib-0043] Oliver, J. K. , Berkelmans, R. , & Eakin, C. M. (2018). Coral bleaching in space and time. In M. J. H. van Oppen , & J. M. Lough (Eds.), Coral bleaching: Patterns, processes, causes and consequences (pp. 27–49). Springer.

[ece39000-bib-0044] Pelosi, J. , Eaton, K. M. , Mychajliw, S. , terHorst, C. P. , & Coffroth, M. A. (2021). Thermally tolerant symbionts may explain Caribbean octocoral resilience to heat stress. Coral Reefs, 40, 1113–1125. 10.1007/s00338-021-02116-8

[ece39000-bib-0045] Pettay, D. T. , & Lajeunesse, T. C. (2007). Microsatellites from clade B Symbiodinium spp. specialized for Caribbean corals in the genus Madracis. Molecular Ecology Notes, 7, 1271–1274.

[ece39000-bib-0046] Petzoldt, T. (2019). Growthrates: Estimate growth rates from experimental data. R Package version. Available online at: https://www.rdocumentation.org/packages/growthrates/versions/0.8.1

[ece39000-bib-0047] Pierangelini, M. , Thiry, M. , & Cardol, P. (2020). Different levels of energetic coupling between photosynthesis and respiration do not determine the occurrence of adaptive responses of Symbiodiniaceae to global warming. New Phytologist, 228, 855–868. 10.1111/nph.16738 32535971 PMC7590187

[ece39000-bib-0049] Quigley, K. M. , Baker, A. C. , Coffroth, M. A. , Willis, B. L. , & van Oppen, M. J. H. (2018). Bleaching resistance and the role of algal endosymbionts. In M. J. H. van Oppen & J. M. Lough (Eds.), Coral bleaching: Patterns, processes, causes and consequences (pp. 111–151). Springer International Publishing.

[ece39000-bib-0050] Rädecker, N. , Pogoreutz, C. , Gegner, H. M. , Cárdenas, A. , Roth, F. , Bougoure, J. , Guagliardo, P. , Wild, C. , Pernice, M. , Raina, J.‐B. , Meibom, A. , & Voolstra, C. R. (2021). Heat stress destabilizes symbiotic nutrient cycling in corals. Proceedings of the National Academy of Sciences, 118, e2022653118. 10.1073/pnas.2022653118 PMC7865147 33500354

[ece39000-bib-0051] Rädecker, N. , Pogoreutz, C. , Voolstra, C. R. , Wiedenmann, J. , & Wild, C. (2015). Nitrogen cycling in corals: The key to understanding holobiont functioning? Trends in Microbiology, 23, 490–497. 25868684 10.1016/j.tim.2015.03.008

[ece39000-bib-0052] Ravelo, S. F. , & Conaco, C. (2018). Comparison of the response of in hospite and ex hospite Symbiodinium to elevated temperature. Marine and Freshwater Behaviour and Physiology, 51, 93–108. Taylor & Francis.

[ece39000-bib-0048] R Core Team (2020). R: A language and environment for statistical computing. R Foundation for Statistical Computing. https://www.R‐project.org/

[ece39000-bib-0053] Reusch, T. B. H. , Ehlers, A. , Hammerli, A. , & Worm, B. (2005). Ecosystem recovery after climatic extremes enhanced by genotypic diversity. Proceedings of the National Academy of Sciences of the United States of America, 102, 2826–2831. 10.1073/pnas.0500008102 15710890 PMC549506

[ece39000-bib-0054] Santos, S. R. , Gutierrez‐Rodriguez, C. , & Coffroth, M. A. (2003). Phylogenetic identification of symbiotic dinoflagellates via length heteroplasmy in domain V of chloroplast large subunit (cp23S)‐ribosomal DNA sequences. Marine Biotechnology, 5, 130–140. 10.1007/s10126-002-0076-z 12876648

[ece39000-bib-0055] Schweitzer, J. A. , Fischer, D. G. , Rehill, B. J. , Wooley, S. C. , Woolbright, S. A. , Lindroth, R. L. , Whitham, T. G. , Zak, D. R. , & Hart, S. C. (2011). Forest gene diversity is correlated with the composition and function of soil microbial communities. Population Ecology, 53, 35–46. 10.1007/s10144-010-0252-3

[ece39000-bib-0056] terHorst, C. P. , Zee, P. C. , Heath, K. D. , Miller, T. E. , Pastore, A. I. , Patel, S. , Schreiber, S. J. , Wade, M. J. , & Walsh, M. R. (2018). Evolution in a community context: Trait responses to multiple species interactions. The American Naturalist, 191(3), 368–380. The University of Chicago Press.

[ece39000-bib-0057] Thompson, J. N. (1998). Rapid evolution as an ecological process. Trends in Ecology & Evolution, 13, 329–332. 10.1016/S0169-5347(98)01378-0 21238328

[ece39000-bib-0058] van Hooidonk, R. , Maynard, J. , Tamelander, J. , Gove, J. , Ahmadia, G. , Raymundo, L. , Williams, G. , Heron, S. F. , & Planes, S. (2016). Local‐scale projections of coral reef futures and implications of the Paris Agreement. Scientific Reports, 6, 39666. 10.1038/srep39666 28000782 PMC5175274

[ece39000-bib-0059] van Oppen, M. J. H. , Oliver, J. K. , Putnam, H. M. , & Gates, R. D. (2015). Building coral reef resilience through assisted evolution. Proceedings of the National Academy of Sciences of the United States of America, 112, 2307–2313. 10.1073/pnas.1422301112 25646461 PMC4345611

[ece39000-bib-0060] Violle, C. , Enquist, B. J. , McGill, B. J. , Jiang, L. , Albert, C. H. , Hulshof, C. , Jung, V. , & Messier, J. (2012). The return of the variance: Intraspecific variability in community ecology. Trends in Ecology & Evolution, 27, 244–252. 10.1016/j.tree.2011.11.014 22244797

[ece39000-bib-0061] Wallingford, P. D. , Morelli, T. L. , Allen, J. M. , Beaury, E. M. , Blumenthal, D. M. , Bradley, B. A. , Dukes, J. S. , Early, R. , Fusco, E. J. , Goldberg, D. E. , Ibáñez, I. , Laginhas, B. B. , Vilà, M. , & Sorte, C. J. B. (2020). Adjusting the lens of invasion biology to focus on the impacts of climate‐driven range shifts. Nature Climate Change, 10, 398–405. 10.1038/s41558-020-0768-2

[ece39000-bib-0062] Williams, S. E. , Shoo, L. P. , Isaac, J. L. , Hoffmann, A. A. , & Langham, G. (2008). Towards an integrated framework for assessing the vulnerability of species to climate change. PLoS Biology, 6(12), e325. 10.1371/journal.pbio.0060325 PMC2605927 19108608

[ece39000-bib-0063] Zilber‐Rosenberg, I. , & Rosenberg, E. (2008). Role of microorganisms in the evolution of animals and plants: The hologenome theory of evolution. FEMS Microbiology Reviews, 32, 723–735. 10.1111/j.1574-6976.2008.00123.x 18549407

