# Supplementary material for: Individual variation in growth and physiology of symbionts in response to temperature
Source: Ecol Evol. 2022 Jun 7;12(6):e9000. doi: 10.1002/ece3.9000 (PMC9173866; doi:10.1002/ece3.9000)
Supplement: Supplementary file 1 — Fig S1‐S3 [file ECE3-12-e9000-s001.docx]

**Supplemental Material**

**Results that include Genotype G3**

At the end of the experiment, genetic analyses indicated that each culture contained only the genotype we expected, except for cultures of G3, which contained both G1 and G3. The extent of contamination suggests that the stock culture of G3 likely contained both G1 and G3. We do not know the relative abundances of genotypes in these cultures, so observed results may be driven largely by either G1 or G3, or a combination of the two.

All genotypes had positive growth rates at each temperature. However, different genotypes had different growth rate responses to increasing temperature (Temperature*Genotype: F_8,60_ = 2.51, P = 0.020). Although every genotype experienced the highest mean growth rate at 30° C, the extent to which growth rates dropped at 32° C varied among genotypes. For example, G1 and G4 showed a steep decline in growth rate at the highest temperature, but G2 and G3 showed little decline (Fig. 1A). Similarly, the extent to which growth rate increased between 26° C and 30° C varied among genotypes, with sharp increases observed for G1, G4, and G5, but little difference observed for G2 and G3 (Fig. 1A). Although carrying capacity tended to be highest at 26° C, we observed more variable responses among genotypes in the response of carrying capacity to temperature (Temperature*Genotype: F_8,60_ = 8.30, P < 0.001). Some genotypes (G3 and G4) showed a steady decline in K with increasing temperature (Fig. 1B). G1 showed a peak in K at 30° C, G1 and G2 showed a decrease in K at 32° C, but G5 showed relatively little variation with temperature (Fig. 1B).

All genotypes demonstrated the ability to acclimate their physiology to each temperature to some extent, but these responses did not reveal a strong effect of historical temperature. Genotypes had different respiration responses at different temperatures (Temperature*Genotype: F_8,59_ = 5.82, P < 0.001). One genotype (G5) showed little variation in respiration across temperatures. The remaining four genotypes showed decreases in respiration at higher temperatures (30° and 32° C) relative to 26° C, but the magnitude of the decrease varied among genotypes (Fig. 2A). Temperature also had a significant effect on gross photosynthesis (F_2,59_ = 6.78, P = 0.002), and genotypes differed in gross photosynthetic rate (F_4,59_ = 24.7, P < 0.001), but, in contrast to the respiration results, there was no significant difference among genotypes in response to increasing temperature (Temperature*Genotype: F_8,59_ = 0.648, P = 0.734). Gross photosynthetic rate increased with increasing temperature, and genotypes G2, G4, and G5 tended to have higher photosynthetic rates than G1 and G3 (Fig. 2B). Patterns of net photosynthetic rate were similar to those for gross photosynthesis; temperature (F_2,60_ = 20.5, P < 0.001) and genotype (F_4,60_ = 31.4, P < 0.001) had a significant effect on net photosynthesis, but again, there was no significant interaction between temperature and genotype (F_8,60_ = 1.12, P = 0.360, Fig. 3).
